# Induction of cytotoxic T lymphocytes primed with Tumor RNA-loaded Dendritic Cells in esophageal squamous cell carcinoma: preliminary step for DC vaccine design

**DOI:** 10.1186/1471-2407-10-261

**Published:** 2010-06-07

**Authors:** Mehran Gholamin, Omeed Moaven, Moein Farshchian, Mahmoud Mahmoudi, Mojtaba Sankian, Bahram Memar, Mohammad Naser Forghani, Reza Malekzadeh, Mohammad Taghi Rajabi-Mashhadi, Mohammad Reza Abbaszadegan

**Affiliations:** 1 Division of Human Genetics, Immunology Research Center, Avicenna Research Institute, Mashhad University of Medical Sciences (MUMS), Mashhad, Iran; 2 Department of Molecular Immunology, Immunology Research Center, MUMS, Mashhad, Iran; 3 Department of Immunobiochemistry, Immunology Research Center, MUMS, Mashhad, Iran; 4 Department of Pathology, Omid Hospital, MUMS, Mashhad, Iran; 5 Digestive Disease Research Center, Tehran University of Medical Sciences, Tehran, Iran; 6 Department of Surgery, Omid Hospital, MUMS, Mashhad, Iran

## Abstract

**Background:**

Dendritic Cells (DC) are potent antigen presenting cells with the ability to prime naïve T cells and convert them to cytotoxic T-lymphocytes (CTL). We evaluated the capability of autologous DCs transfected with total tumor and normal RNA to induce cytotoxic CTL as the preliminary step to design a DC-based vaccine in the esophageal squamous cell carcinoma (ESCC).

**Methods:**

Monocytes-derived DCs were electroporated with either total tumor RNA or normal RNA. T cells were then primed with tumor RNA transfected DCs and lytic effects of the generated CTL were measured with Cytotoxicity assay and IFN-γ Release Elispot assay.

**Results:**

Cytotoxicity was induced against DCs loaded with tumoral RNA (%24.8 ± 5.2 SEM) while in normal RNA-loaded DCs, it was minimal (%6.1 ± 2.4 SEM) and significantly lower (p < 0.05). INF-γ secretion was more than 2-folds higher in tumoral RNA-loaded DCs when compared with normal RNA-loaded DCs (p < 0.05).

**Conclusion:**

Electroporating DCs with tumor RNA generated tumor antigen presenting cells which in turn enhanced cytotoxic effects of the T cells against ESCC. This may be a useful autologous *ex vivo *screening tool for confirming the lytic effects of primed T cells on tumors and evaluate probable further adverse effects on noncancerous tissues. These data provide crucial preliminary information to establish a total tumor RNA-pulsed DC vaccine therapy of ESCC.

## Background

Gastrointestinal cancers are the most frequent cancer malignancy in the Iranian population with substantial numbers of the patients reported from northern and northeastern Iran. Esophageal squamous cell carcinoma (ESCC) is the fifth leading cause of cancer-related deaths in Iran [1]. Most cases of ESCC in Iran as well as many other countries are diagnosed in advanced stages [2]. Surgical treatment and adjuvant therapeutic modalities such as chemo- and radiotherapy have only a minimal effect on patient outcome and the overall 5-years survival rate is less than 25%.

Immune system plays an important role in counterbalancing against the malignant transformation and escape from immunologic surveillance and the subsequent immune privilege of tumor may account for the development of some cancers including ESCC [3]. On the other hand, the suggested correlation between the infiltration of T cells in the tumor environment and good prognosis demonstrated in several tumors represents a naturally occurring adaptive immunity in cancer patients [4]. Immunotherapeutic strategies are designed and optimized based on a better understanding of different escape mechanisms. Nonfunctional presentation of tumoral antigen and further suppression of an effective immune response is one of the most important mechanisms of immune evasion which has been targeted in designing a number of immunotherapeutic modalities.

Dendritic cells (DC) are professional antigen capturing cells which initiate primary and further secondary immune responses *in vivo*. Their unique functions are mediated by priming the naïve T cells and cross-presenting various antigens. Maturation is an important characteristic of DCs during which immature DCs, as phagocytic cells capture foreign antigens at the site of injury and become mature cells along the process of presenting the antigen peptides on MHC class I and II molecules [5]. Mature DCs will then migrate to local lymph nodes to present antigens to naïve T cells and convert them into effector cells of the immune system against non-self antigens [6]. Taking advantage of this *in vivo *model, incubation of DCs with tumor specific molecules such as proteins, peptides, or lysates, or transfecting them with nucleic acids encoding tumor specific antigens is the basic concept of designing DC-based cancer immunotherapy [7].

Electroporation is assumed as the most efficient method for delivering the genetic materials into the cells [8]. In this procedure, a mixture of cells and mRNA are pulsed at a specific voltage for a short period of time. Resulting is a small pore on the cell membrane which makes it possible to pass mRNA into the cells [9]. Gilboa et al pulsed DCs with tumor antigen-encoding mRNA and reported an effective therapeutic tumor vaccination when it was administered in a murine model for the first time [10]. Although various studies have tried to find a specific tumor marker for ESCC, an appropriate marker has yet to be found for the purpose of immunotherapy. Therefore, in this study we loaded DCs with total mRNA to transfer the genetic material. T cell response was evaluated after priming with DCs transfected with tumoral mRNA from ESCC patients as a preliminary step to establish a DC vaccine for ESCC.

## Methods

### Sample collection and RNA isolation

Four histologically-approved ESCC patients were included in the study prior to any therapeutic interventions. After obtaining informed consents, approximately 50 ml of blood was collected in heparinized vials for extraction of peripheral blood mononuclear cells (PBMCs). Tumoral tissue was microdissected and stored in RNAlater (Qiagen, Hilden, Germany) until RNA extraction. Corresponding normal tissue from the affected esophagus was obtained using the same protocol. Total RNA was extracted by RNeasy kit (Qiagen, Hilden, Germany). RNA integrity was confirmed by electrophoresis on agarose gel and the concentration and purity was checked by spectrophotometry at 260 nm and 280 nm. The study was approved by the Research Ethics Committee of Mashhad University of Medical Sciences.

### Generation of dendritic cells

Peripheral blood mononuclear cells were obtained from 50 ml of heparinized whole blood via Ficoll-Hypaque (Biosera Inc., East Sussex, United Kingdom). The generation of DCs was performed on CD14^+ ^cells isolated by the EasySep^® ^Human Monocyte Enrichment Kit without CD16 Depletion (Stemcell Technologies, Vancouver, Canada). Cells were seeded for 5 days into six-well plates in RPMI1640 supplemented by 10% heat-inactivated FBS, penicillin (100 U/ml), streptomycin (100 mg/ml), L-glutamine (2 mM), sodium pyruvate, 2ME (2-Mercaptoethanol) (50 mM), human Granulocyte Monocyte-Colony Stimulating Factor (GM-CSF) 800 IU (R&D System Inc., Minneapolis, MN, USA) and IL-4 500 IU (R&D, USA). GM-CSF and IL-4 were again added on the third day. The production of immature DCs was detected by specific CD markers using BD FACSCalibur (BD Biosciences, CA, USA). Cells were electroporated with tumor or normal mRNA on the sixth day, followed by the maturation of the cultured DCs with TNF-α (50 ng/ml) 4 hours after electroporation. Electroporated mature cells were analyzed for transfection using flow cytometry after 24-48 hours.

### FACS (Fluorescence-Activated Cell Sorting) analysis of immature DC

Immunofluorescence staining of monocyte-derived DC with differentiation and activation markers was performed for the phenotypic analysis of DCs. Anti-HLA DR, CD1a, CD80, CD86, CD83, FITC-conjugated (Fluorescein Isothiocyanate), and CD14 PE-conjugated (Phycoerytherin) mouse monoclonal antibodies (IQ Company, Netherlands) were used for the FACS analysis. Appropriate mouse PE/FITC isotype was used as negative control.

### RNA electroporation

Prior to electroporation on day 6, immature DCs were washed twice with the Opti-MEM medium (GibcoBRL, Eggenstein, Germany) and resuspended to a final concentration of 10 × 10^6 ^cells/ml. Subsequently, 100 μl of the cell suspension was initially mixed with 2.5 μg of Enhanced Green Fluorescent Protein (EGFP) mRNA per 10^6 ^cells to confirm the transfection procedure. After confirming the transfection procedure, DCs were transfected with 1.5 μg of tumoral mRNA per 10^6 ^cells or 1.5 μg of corresponding normal mRNA per 10^6 ^cells. Transfection was performed using a BTX ECM830 square-wave electroporator with a single 500 V/300 μs pulse (Genetronics, San Diego, CA, USA).

After pulsing, all the cells were gently and immediately removed from the cuvette and poured into 6 well plates, in which the containing media was pre-warmed at 37°C. For negative control of electroporation reaction, mock DCs were electroporated under the same conditions but without the addition of mRNA.

### Generation of in vitro-transcribed EGFP mRNA

In order to confirm the gene transfer efficiency by the electroporation method, *in vitro*-transcribed (IVT) EGFP mRNA was applied as a reporter gene. pGEM4Z/GFP/A64 plasmid containing the poly(A) template (kindly provided by Dr. E. Gilboa, University of Miami, FA, USA) was utilized for *in vitro *transcription of EGFP mRNA. This plasmid was transformed into E. coli Top10F and purified with a QIAprep Spin Miniprep Kit (Qiagen, Hilden, Germany). Following vector linearization by the restriction enzyme SpeI and purification, DNA template was used for *in vitro *transcription under the control of a T7 promoter with the T7 RNA polymerase. *In vitro *capped RNA was synthesized by the Message MACHINE kit (Ambion, Austin, TX, USA) according to the manufacturer's instructions. The concentration and purity of amplified EGFP RNA was evaluated by spectrometric analysis of absorbance at 260 (A260) and 280 nm (A280) and by 2% agarose/EtBr gel electrophoresis. The DCs electroporated with IVT EGFP mRNA were directly checked by fluorescent microscope and underwent FACS analysis to evaluate the efficiency of transfection which is represented by the percentage of transfected cells in a live population. The introduction efficacy is calculated by multiplying the percentage of viable cells by the transfection efficiency[11].

### Induction of tumor-specific CTL using DCs transfected with tumor RNA

For CTL generation, the T-cell-enriched non-adherent fraction of PBMCs was obtained after the DC plastic adherence step was performed. T-Cells were suspended in RPMI1640 with 10% fetal calf serum, 25 mM Hepes, L-glutamine, and antibiotics. Of the T-cell-enriched PBMCs, 2 × 10^6 ^cells were combined with 2 × 10^5 ^transfected matured DCs and 10 ng/ml IL-7 in a total volume of 2 ml, in the wells of a 24-well tissue culture plate and cultured at 37°C in 5% CO_2 _for 8 days. IL-2 was added to the culture in the third day at a concentration of 20 IU/ml. After 8 days, the effectors were harvested, washed, counted, and re-stimulated with newly transfected DCs. Adding IL-2 and IL-7 with the same concentrations to the culture, the cells were incubated for another 8 days to successfully induce tumor specific CTL.

### Cytotoxicity test

To determine cytotoxicity, the calcein-AM cytotoxicity assay was performed. Prior to the cytotoxicity assay, target cells were re-suspended in RPMI-1640 complete medium at a final concentration of 10^6 ^cells/ml and incubated with 10 μM calcein-AM (Invitrogen inc., Grand Island, NY, USA) for 30 min at 37°C with occasional shaking, treated with 50 μg/ml of mitomycin C for 30 min, and washed three times with RPMI-1640 medium. Effectors and calcein-labeled targets were co-cultured in U bottom 96-well plates in triplicates for 4 h at 37°C in a total volume of 200 μl with various effector:target (E:T) ratios i.e. 1:1, 3:1 and 9:1. Supernatant samples were measured using a fluorescence spectrophotometer (FP-6200, Jasco, Japan; exciting filter: 485 ± 9 nm; band-pass filter: 530 ± 9 nm). Data were expressed as arbitrary fluorescent units (AFU). Percent cytotoxicity of the assay was calculated by the following formula: [(test release - spontaneous release)/(maximum release - spontaneous release)] × 100. The maximum release and spontaneous release represent calcein release from the targets in the medium, with and without 2% Triton X-100, respectively.

### IFN-γ release Elispot assay

The IFN-γ release Elispot assay was performed by an Elispot kit (U-CyTech biosciences, Utrecht, Netherlands) according to the manufacturer's instructions. Briefly, 96-well plates with transparent membranes were coated with 50 μL primary anti-IFN-γ antibodies and incubated at 4°C for at least 18 h. After washing the plates with the washing buffer five times, individual wells were blocked for nonspecific binding with 200 μL RPMI containing 10% FBS at 37°C for 1 h. After removal of the blocking solution, 4 × 10^4 ^responder T cells and 2 × 10^3 ^mitomycin-treated stimulator cells were added in a total volume of 200 μL RPMI medium containing 10% FBS. Stimulator cells included DCs transfected with tumoral or normal mRNA and also mock DCs. Each experimental condition was plated in triplicates and incubated at 37°C in 5% CO_2 _for 24 h. Cultures were removed and the plates were washed ten times with the washing buffer. One hundred microliters of properly diluted biotinylated detection anti-IFN-γ antibody was added to each well and incubated overnight at 4°C. The plates were washed with the washing buffer five times and 50 μl of labeled anti-biotin antibody was added to each well and incubated at 37°C in the dark for 1 h. Washing was repeated five times and activator solutions were added to detect the sites of cytokine secretion by revealing the black spots. The plates were developed in the dark for 30 min. The reactions were stopped by rinsing with tap water and spots were imaged and counted at the final step.

### Statistical analysis

Results were expressed as the mean ± SD. Statistical analysis of cytotoxicity and Elispot tests were conducted by unpaired Student's *t*-test and paired Student *t*-test using SPSS software (version 16). A value of p < 0.05 was considered statistically significant.

## Results

After preliminary optimization and confirming efficient gene transfer in one patient, a total of 3 patients were enrolled in the study. All the patients were histologically diagnosed as esophageal squamous cell carcinoma. Clinicopathological features of the patients are demonstrated in Table 1.

**Table 1:** Clinicopathological characteristics of patients.

Patient	Age	Sex	Tumor Location	Tumor Grade	TNM Classification
**Patient 1**	65	Male	Middle	M.D.	T3N0M0
**Patient 2**	51	Female	Middle	M.D.	T3N0M0
**Patient 3**	79	Male	Middle	M.D.	T3N0M0
**Patient 1**	98	Male	Middle	W.D.	T2N1M0

"M.D." represents moderately differentiated and "W.D." represents well differentiated

### FACS analysis of monocyte-derived DCs

Immuno-phenotyping of the DCs was analyzed five days after monocytes were cultured and required cytokines were added. Specific CD markers including CD1a, CD14, CD80, CD86, CD83, and HLA-DR were detected to confirm the generation of immature DCs. CD14 was decreased to less than 1% and CD1a was increased to the average of approximately 95%, representing transformation of monocytes into DCs. After 2 days, FACS analysis was performed to detect specific CD markers of matured DCs. CD83 was increased to an average of 58.9% confirming the maturation process. Results of the FACS analysis are shown in Table 2.

**Table 2:** Phenotypic characterization of immature and mature DCs by flow cytometry.

	% Immature DC	% Mature DC
**CD14**	0.9	4.7
**CD1a**	94.9	94.6
**CD80**	11.49	39.29
**CD86**	31.37	76.06
**CD83**	10.84	58.94

### Electroporation efficacy represented by GFP mRNA transfection into DC

**Figure 1:**
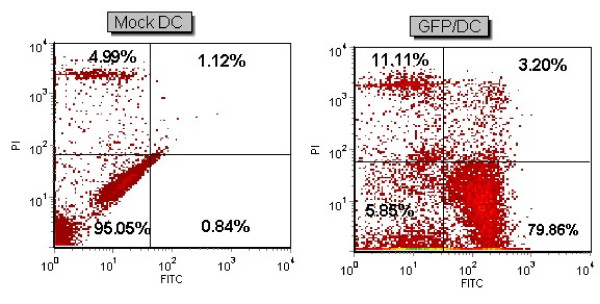
**mRNA transfection of DC**. GFP mRNA delivery into DCs of patient 1 with electroporation (500 V, 300 μs). FACS analysis of transfected DC and Mock as a control is shown. Viability percentage was 85.7% in transfected cells as compared with 93.9% in control Mock. Transfection efficiency was 79.8%.

In order to establish the optimized gene delivery into monocyte-derived DCs, the transfection efficacy of *in vitro *transcribed GFP mRNA into DCs was first confirmed. Successful GFP expression, as a reporter gene, was assessed by fluorescent microscopy and FACS analyses. The average viability percentage of the transfected cell population was 94.5% as compared to 97.9% in mock cells and the average transfection efficiency was 87.8%. The flow cytometry results of patient 1 are presented in Figure 1. The kinetic of GFP expression was analyzed afterwards and an approximately 30% expression was detected 96 h after electroporation.

### Cytotoxicity results

**Figure 2:**
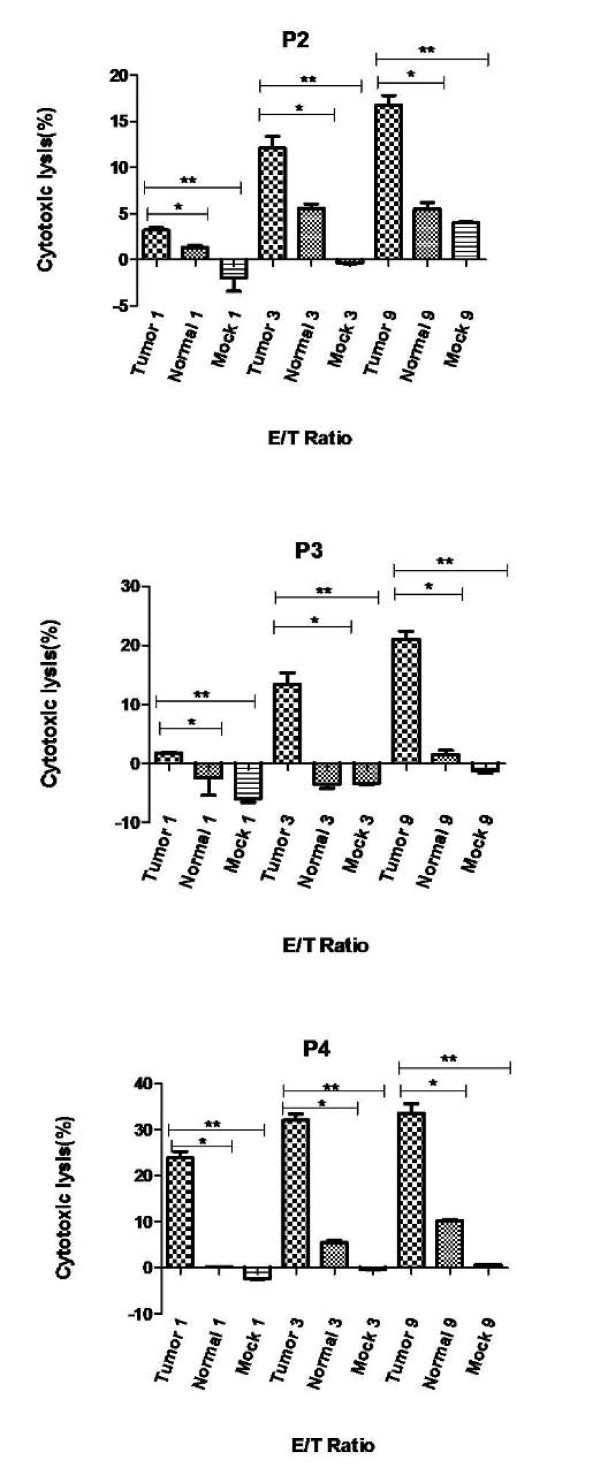
**Cytotoxicity assay results representing cytotoxic activity of CTLs induced by DC/tumor-mRNA**. Cytotoxicity assay was performed against DC/Tumor mRNA, DC/Normal mRNA and DC/Mock as targets at various effector:target (E:T) ratios assessed by Calcein-AM release assay. Experiments were repeated three times, and representative data of similar results are shown. For example, in the patient 3 cytotoxicity against DC/Tumor-mRNA, DC/Normal mRNA and DC/Mock were 22%, 2% and -1% respectively. *p < 0.05 (Tumor vs. Normal); ** p < 0.05 (Tumor vs. Mock).

Cytotoxicity of primed lymphocytes with tumoral mRNA-loaded DCs in ESCC patients were assessed by measuring the cytotoxicity of CTLs against DC targets loaded with tumoral RNA, or normal RNA, or mock DC as negative control. Cytotoxicity percentage against DCs loaded with tumoral RNA ranged from 17.5 to 35.0. In all the patients, significant differences were observed between cytotoxicity against DCs loaded with tumoral RNA and mock DCs (p < 0.05). Cytotoxicity against DCs loaded with tumoral RNA was significantly higher than the corresponding value for DCs loaded with normal RNA for each patient separately and when the average of the two groups were compared with paired *t*-test (24.8 ± 5.2 SEM and 6.1 ± 2.4 SEM, p < 0.05). The Cytotoxicity results of the ESCC patients are shown in Figure 2.

### Elispot results

**Figure 3:**
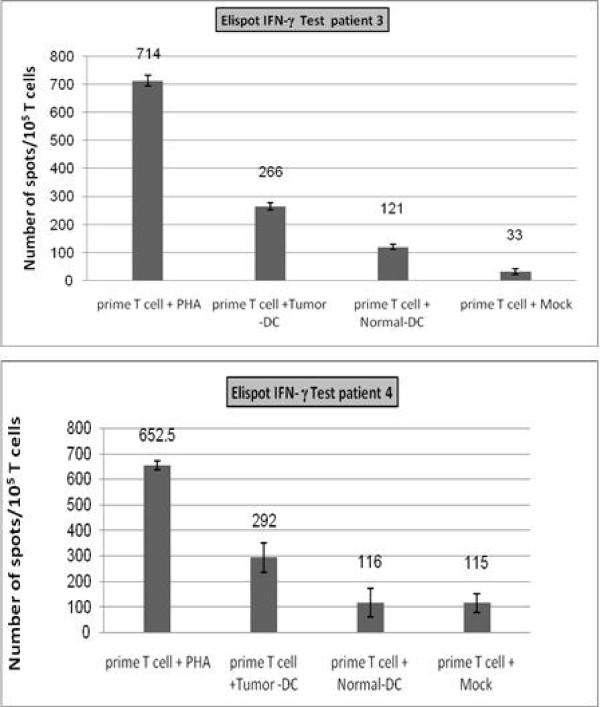
**INF-γ secretion Elispot assay results**. INF-γ spots are counted per 10^5^ T cells. Results are recorded as mean number of INF-γ spots with 95% confidence interval (CI) indicated on the bars. Specific T cell activation and INF-γ secretion was more than 2-folds higher in DC/Tumor-mRNA when compared to DC/Normal-mRNA in both patients (p < 0.05).

Elispot assay was performed to reconfirm the significant differences between tumoral and normal mRNA-loaded DCs observed in the results of cytotoxicity assay. Specific T cell activation and INF-γ secretion was more than 2-folds higher in tumoral RNA-loaded DCs when compared to normal RNA-loaded DCs in patients 3 and 4 (p < 0.05). For patient 2, Elispot assay was not performed due to insufficient amount of DCs (less blood was possible to obtain from the patient) and only cytotoxicity test was performed. The results of the assay are shown in Figure 3.

## Discussion

Due to its asymptomatic nature, ESCC is often diagnosed in its advanced stages where the common therapeutic modalities are not very effective and the 5-year survival rates do not exceed 25%. Therefore, approaches to novel therapeutic modalities seem to be an essential step to improve the survival rate of ESCC patients. We have previously shown that immune escape is a frequent event in the Iranian ESCC patients. This suggests that immunotherapy can be a potentially effective tool to reverse malignant transformation of cells by enhancing immune responses [12].

Dendritic cells have a unique capacity to capture, process, and present antigens that establish primary and secondary immune responses [13-15]. Taking advantage of their capabilities, DC transfection with nucleic acids encoding tumor specific antigens leads to processing and presenting the antigens and consequently, activating tumor-specific CTLs [6]. Considering this fact, DCs are proposed as an effective target for vaccinating cancer patients against tumor antigens and promoting an immune response against tumors [16,17]. It has been shown that RNA transfection of DCs offers numerous advantages over transferring other molecules [18,19]. mRNA delivery system is an efficient method compared to the viral vectors as they do not pose the problems associated with viral vectors [20]. DCs can be introduced by total tumoral mRNA and the identification of tumor associated antigens (TAAs) or HLA-typing is not obligatory [21] especially in cancers such as ESCC in which wide variety of genotypic and phenotypic features lead to lack of specific tumor markers in different populations [20]. Loading total tumoral mRNA decreases the probability of immune escape via polyclonal activation of T cells against varied range of tumor-specific antigens and minimizes the consequences of antigen loss in mutant tumor cell clones. As RNA has a short half-life and lacks the ability to integrate into genome, many problems with viral vectors are not encountered [14]. With regards to gene expression, mRNA transfection is more efficient than DNA transfection as it bypasses the transcriptional regulation processes and has easy access to the cytoplasmic translation machinery when transferred into the cells [22]. Both CD4+ and CD8+ T lymphocytes will be activated by tumoral RNA transfer into DCs [23]. Electroporation has proved to be a more efficient method of gene transfer than many other techniques [22]. Thus, the electroporation method was utilized in order to achieve efficient total mRNA transfer into DCs as the preliminary step of a DC vaccine design for ESCC. In the present study, the results of mRNA transfer efficiency with electroporation (approximately 80%) were comparable to the previous reports of highly efficient transfer applying the same method [5,24].

Clinical trials using antigen-loaded DCs have been published in various types of cancer including myeloid leukemia [25], medullary thyroid carcinoma [26], metastatic melanoma [27], pancreas cancer [28], colorectal cancer [29] and other malignancies. To our knowledge, only Millano et al have reported DC therapy on two ESCC patients in addition to five esophageal adenocarcinomas with some technical variations compared to our study [30]. Our results show that primed T lymphocytes have obtained the capability to lyse the tumor RNA-loaded DCs. The percentage of lysis varies between 17.5 and 35 in the E:T ratio of 9. A serious limitation of this study was restricted amount of DCs since we were not able to perform leukophoresis in our patients. Many studies have performed the cytotoxicity assay in higher ratios [31,32]. However, due to our restrictions in the available amount of DCs, we did not perform the assay in higher ratios and probably in higher E:T ratios, greater percentage of cytotoxicity might have been observed. Cytotoxicity percentage in the mock DCs, as in controls, was negligible.

Transfecting DCs with total tumor RNA brings up a major concern of a consequent adverse effect through breaking tolerance towards self antigens and thus destructing effects on normal tissues as well. In the present study, cytotoxicity against normal RNA-loaded DCs was minor while lytic activity of primed T cells induced by DCs loaded with tumor RNA was significantly higher than normal RNA-loaded DCs in each patient and when comparing the average of both groups by paired *t*-test. Millano et al have reported enhanced lytic response against some of the studied normal tissues in esophageal adenocarcinoma suggesting the possibility of adverse effects against normal tissues in esophageal cancer [30]. Therefore, a preclinical *ex vivo *autologous assessment may be a wise step to include the beneficiary cases and predict the possibility of the adverse effects.

## Conclusion

In summary, we demonstrated that electroporating DCs with tumor mRNA will enhance the cytotoxic effects of T cells against tumor in ESCC patients. Enhanced cytotoxicity induced by tumor RNA-loaded DCs is introduced as a useful autologous *ex vivo *screening tool for confirming the lytic effects of primed T cells on tumor and evaluates probable further adverse effects on noncancerous tissues. Enhancing cytotoxicity against tumoral cells is a crucial preliminary step to establish a total tumor RNA-pulsed DC vaccine therapy of ESCC.

## Competing interests

The authors declare that they have no competing interests.

## Authors' contributions

MG participated in the study design, carried out the experimental assays, analyzed the data and participated in drafting the manuscript. OM drafted the manuscript, participated in patient collection and carried out statistical analysis and data interpretation. MF participated in the experimental assays and data analysis. MM and MS participated in the study design, coordination and data interpretation. BM carried out data collection and tissue preparation. RM, MTRM and MNF participated in sample and data collection and study coordination. MRA participated in study design and coordination, data interpretation and scientific revision of the manuscript. All authors read and approved the final manuscript.

## Pre-publication history

The pre-publication history for this paper can be accessed here:

http://www.biomedcentral.com/1471-2407/10/261/prepub
